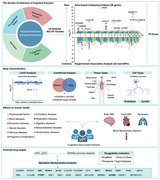# Large‐scale exome sequencing identifies novel genes for socioeconomic status, education, and cognition

**DOI:** 10.1002/alz.088059

**Published:** 2025-01-03

**Authors:** XinRui Wu, Jin‐Tai Yu

**Affiliations:** ^1^ Huashan Hospital, State Key Laboratory of Medical Neurobiology and MOE Frontiers Center for Brain Science, Fudan University, Shanghai China; ^2^ Huashan Hospital, Fudan University, Shanghai, Shanghai China

## Abstract

**Background:**

Cognition and its two critical proxies, socioeconomic status (SES) and educational attainment (EA), contribute substantially to human health and are heritable. Elucidating the genetic characteristics of SES/EA/Cognition not only helps to understand the innate individual differences in cognition, but also aids in unraveling the biological mechanisms of complex cognitive‐related disorders such as Alzheimer’s disease (AD). Here, we explored the rare and common protein‐coding variants impacting the comprehensive cognition phenotypic spectrum by leveraging large‐scale exomes.

**Method:**

Using 350,770 exomes from the UK Biobank, we performed gene‐based collapsing analysis and single‐variant analysis for rare and common variants, respectively. Identified genes were biological annotated at multiple levels of gene sets, tissue types and cell types. The identified genetic associations with SES/EA/Cognition were further extended to brain structure, blood chemical markers, and neuropsychiatric phenotypes. Lastly, Mendelian randomization (MR) analysis and druggability evaluations were performed to reveal potential therapeutic targets.

**Result:**

We identified 79 SES/EA/Cognition‐associated genes (64 novel) after Bonferroni correction, 29 from gene‐based collapsing tests for rare variants (*P* < 2.19 × 10^‐8^) and 50 from single‐variant association analysis for common variants (*P* < 1.08 × 10^‐7^). The intersection of rare variants with genome‐wide association studies signals prioritized *ADGRB2* and *KDM5B*, and the effect of rare *KDM5B* variants was independent of nearby common variants. Pathway enrichment analysis revealed the involvement of synapse and hepatic metabolism. Multiple human brain tissue types as well as cell clusters of excitatory neuron and microgila were enriched. Rare variants in five genes (*C2CD2L*, *IGF1R*, *AKNRD12*, *GIGYF1*, *KDM5B*) and 35 common variants showed pleiotropy with broader biological phenotypes, including hippocampus volume and neurodegenerative diseases. MR analysis revealed the causal relationships between the expression levels of 23 genes in brain with SES/EA/Cognition (7 mapped to drug targets).

**Conclusion:**

This study utilizes large‐scale exome sequencing to provide a more comprehensive genetic framework underlying SES/EA/Cognition, offering informative associated genes and biological insights. The identified 79 genes across full allele frequency spectrum, 64 of which exhibited novel associations, would provide an abundant resource for further in‐depth molecular mechanistic studies and drug development for cognition.